# Tuberculosis transmission at healthcare facilities in India

**DOI:** 10.4103/0970-2113.48893

**Published:** 2009

**Authors:** Ashutosh N. Aggarwal

**Affiliations:** *Department of Pulmonary Medicine, Postgraduate Institute of Medical Education and Research, Chandigarh, India. E-mail: ashutosh@indiachest.org*

Tuberculosis is an important public health problem in India, and considerable effort is being devoted to the diagnosis and management of this condition in an attempt to lower the overall disease burden in the community. Clinicians and other paramedical staff involved in diagnosis and care of these patients are likely to be frequently exposed to live mycobacteria. Although the issue has never been openly or seriously discussed, there is need to be aware of these risks. Two important considerations need attention. First, is nosocomial transmission of tuberculosis likely, and if so, why? Second, does this problem occur in India, and if so, in what magnitude?

In general, the prevalence of tuberculosis among patients visiting a general healthcare facility is considerably higher than that in the general population. It therefore stands to logic that the probability of nosocomial transmission of tuberculosis is higher than the transmission occurring in the general population. In fact, several old studies conducted 60–80 years ago in the West had shown higher frequency of tuberculin positivity, tuberculin conversion, and cases of tuberculosis among nurses caring for these patients.^1^ These observations were also supplemented by animal studies, and inoculation of guinea pigs with dust from rooms frequented by medical students led to death of these animals due to tuberculosis.^1^ Clearly, these data reflect the increased exposure risk to healthcare workers. In India, this risk is further increased by the extremely large load of ‘open’ cases visiting the hospitals. Delays in diagnosis and treatment often prolong the infectious phase for these patients. Suboptimal treatment (improper drug regimens, poor adherence to treatment, limited drug availability, etc.) often compounds this problem, further increasing the risk as each patient makes multiple visits to the laboratory or outpatient department. The Revised National Tuberculosis Control Program has successfully addressed these key issues.^2^ However, gaps still remain for patients receiving treatment outside the program. Poor infection control measures and deficiencies in knowledge and awareness are two other important additional factors responsible for nosocomial transmission.^3^ Infection control measures are grossly inadequate at most healthcare facilities, especially at the primary care level. Facilities to isolate infectious patients admitted to hospitals are scarce. Both the outpatient and inpatient settings are overcrowded, with an unintentional clustering of patients and those susceptible to the disease. Provisions for adequate ventilation and sunlight, two important measures that can take care of mycobacteria present in the environment, are usually lacking. In addition, personal protective equipments for healthcare workers (for instance, masks) are not usually available. The problem is compounded by gaps in knowledge and awareness of both patients and healthcare providers. In general, there is total lack of information and awareness about transmission in healthcare facilities in India. Most healthcare workers believe that such transmission is an unavoidable occupational hazard, and do not even take basic necessary precautions while handling infectious clinical material. This is partly also related to absence of education on occupational safety and hygiene in medical curriculum. The patients themselves lack good cough etiquette in most instances, as hardly any counseling is provided to them. They also often lack knowledge of basic sputum disposal techniques.

All these data suggest that nosocomial transmission of tuberculosis is an issue that simply cannot be wished away. The concerns are even more valid in our country. India has more tuberculosis patients than any other country, and the probability of nosocomial transmission is expected to be proportionately higher. Despite this, there is very little information on the subject, although some recent studies have attempted to provide some data that largely center on transmission among healthcare workers.^3^ Preliminary information has been gathered for incidence and prevalence of both infection and disease, and some experimental data and risk factor analysis are also available.

We recently published our experience regarding the risk and incidence of active tuberculosis among resident doctors at our institute.^4^ The study had two groups. The first included 538 resident doctors already working at our institute, of whom 470 were interviewed. The second included 235 resident doctors freshly admitted to the institute over a one-year period, of whom 231 were prospectively followed up. Tuberculosis was diagnosed based on history, radiology, laboratory workup, and response to treatment. In the first group, nine doctors had tuberculosis while working in hospital, amounting to 11.2 new cases per 1000 person-years of exposure. Six of them had extrapulmonary disease. Seven worked in medical and two in surgical specialties and none had any comorbidity or other risk factors. In the second group, four doctors (two junior and two senior residents) developed active tuberculosis in the first year of their work at our institute, amounting to an overall incidence of 17.3 per 1000. Three of them had extrapulmonary disease, and the fourth had sputum positive pulmonary disease. Again none had any comorbidity or other risk factors. These data suggested high rates of active disease among physicians in training, with incidence figures much higher than those in general population. A notable point was that most had extrapulmonary disease (and were therefore diagnosed on clinical rather than microbiological criteria), and it is possible that they suffered from primary rather than reactivation disease. It was however not clear if infection was acquired during or before stay at our institute.

Around the same time, a ten-year review of hospital records was published from Vellore.^5^ Data on 125 healthcare workers who received treatment for tuberculosis during 1992–2001 were collected. Incidence of pulmonary tuberculosis varied from 0.37–1.57 per 1000, and that of extrapulmonary tuberculosis varied from 0.34–1.57 per 1000, in different years. Overall incidence of sputum positive disease was thus quite similar to general population, whereas that of extrapulmonary tuberculosis was higher than general population. The maximum cases were diagnosed among nursing staff and students. Certain groups of healthcare workers had higher incidence in certain years, suggesting focal outbreaks, although this could not be verified from this retrospective analysis.

There is also some information on latent tuberculosis among healthcare workers. A cross-sectional prevalence study was conducted at Sevagram on 726 healthcare workers with no prior history of tuberculosis.^6^ The cohort comprised mainly of medical students (31%), nursing students (17%), and nurses (22%). Both tuberculin skin testing and an ELISA-based gamma-interferon release assay were used. Fifty percent healthcare workers were found latently infected (positive by either of the two tests). Nurses, nursing students, orderlies, and laboratory staff showed higher prevalence of infection, and advancing age and employment duration were identified as risk factors. However, these prevalence figures are likely to be an underestimate due to nonparticipation by the vast majority of senior physicians at the hospital. All these healthcare workers were kept under follow up and a repeat survey of 216 medical and nursing students of this cohort was conducted after a gap of 18 months.^7^ Both tuberculin skin testing and gamma-interferon release assay were again performed in an attempt to estimate annual risk of infection. Data from 147 valid follow-ups with both tests were reported. Eleven (7.5%) healthcare workers had conversion on one or both tests; this corresponds to annual risk of infection of approximately 5%. If average community based annual risk of infection in India is considered about 1.5%, the 3.5% excess risk among healthcare workers may be attributable to nosocomial exposure.

Molecular epidemiology tools have also been used to describe small-scale nosocomial transmission among patients admitted to two wards at a tuberculosis hospital in Delhi.^8^ DNA fingerprinting was carried out on 83 *Mycobacterium tuberculosis* isolates. Eight of these strains were grouped into three clusters on the basis of identical DNA fingerprints. Within each cluster, epidemiologic data showed overlapping hospitalization period, suggesting patient-to-patient transmission during hospital stay.

Two recent systematic reviews have collated available information on the topic, including that available form India, to quantify risk of tuberculosis among healthcare workers.^9^,^10^ The methods used to derive these estimates are based on several assumptions, and figures thus reported are therefore imprecise and variable. For instance, the risk of tuberculosis disease among healthcare workers attributable to nosocomial transmission is calculated as 42 and 1092 based on data from two different studies.^9^ Despite these shortcomings, the analysis does suggest that the magnitude of this problem is considerable in India. The existing healthcare infrastructure and infection control measures are ill equipped to deal with this colossal problem, and urgent remedial measures are necessary.
